# Impact of Superplasticizers on the Performance of Low-Grade Limestone-Based Cement Mixes

**DOI:** 10.3390/ma17112500

**Published:** 2024-05-22

**Authors:** Murugan Muthu, Boddapati Ganesh Kumar, Neven Ukrainczyk, Łukasz Sadowski, Eddie Koenders

**Affiliations:** 1 Department of Materials Engineering, Wroclaw University of Science and Technology, 50-372 Wrocław, Poland; lukasz.sadowski@pwr.edu.pl; 2 Larsen & Toubro Construction Research and Testing Center, Chennai 600 089, India; bgkk@lntecc.com; 3 Institute of Construction and Building Materials, Technische Universität Darmstadt, 64287 Darmstadt, Germany; ukrainczyk@wib.tu-darmstadt.de (N.U.); koenders@wib.tu-darmstadt.de (E.K.)

**Keywords:** low-grade limestone, cement, gypsum, superplasticizers, marsh cone test

## Abstract

Low-grade limestone (LGL) is not used to produce cement clinker, but this leftover material in cement quarries increases the water demand when used as a filler in concrete production. In this study, the effect of six commercial superplasticizers on the performance of cement mixes containing 35% LGL and 2% gypsum was investigated. The optimal doses of these superplasticizers were found in a range of different water/binder (w/b) ratios by conducting several Marsh cone and mini-slump tests. The addition of a superplasticizer with a higher active solid content produced a maximum cement flow, regardless of the w/b ratios. The LGL-based mortar samples admixed with this superplasticizer obtained a maximum compressive strength of about 36 MPa at the end of 28 days. SEM and XRD results showed the formation of a new calcium-rich mineral in their microstructure. These findings highlight the impact of the type and properties of superplasticizers on the performance of concrete mixes containing LGL as a supplementary cementitious material.

## 1. Introduction

Electricity and heat production, agriculture, forestry and transportation, industry, and buildings are the main economic sectors causing global anthropogenic greenhouse gas emissions [1]. The cement industry is one of the main contributors to industrial CO_2_ emissions, accounting for approximately 8% of the global CO_2_ emissions [2]. The use of industrial by-products, including fly ash and slag, in concrete mixes reduces CO_2_ emission by up to 0.81 kg/kg of cement [3], but its availability is limited and cannot meet global demand [4]. The fly ash content is limited to 30% in most concrete mixes. The problem behind the use of high-volume fly ash in concrete mixes is the delay in setting and the slower rate of strength gain at the beginning of age [5]. Slag can be substituted for up to 70% clinker; however, its availability is inadequate [6]. Therefore, it is necessary to identify new sources of inorganic materials as partial cement replacements to protect the environment and sustainability.

Some non-reactive or weakly reactive powders, such as marble [7], granite [8], and limestone [9], showed promising contributions. Among these cement replacement materials, limestone powder shows great potential due to its very low embedded CO_2_ emission, abundant reserve on Earth, and low cost [10]. Limestone reserves are abundant on Earth and up to 35% are recommended in the production of concrete mixes [11]. They are generally divided into high- and low-grade grades based on their CaO content [12]. Low-grade limestone (LGL) contains 38% CaO and is generally not considered in the production of cement clinker. This leftover material as a replacement for cement can greatly reduce cost and CO_2_ emissions. In general, the addition of limestone accelerates the hydration of calcium silicates as a result of its nucleation effect and promotes the formation of calcium silicate hydrate (CSH) [13]. The nucleation of the CSH gel on limestone surfaces precipitates heterogeneously, but this precipitation facilitates the reduction in the volume of the open capillary pores and partially compensates for the dilution caused by the filler material [14]. The dissolution of limestone also releases calcium ions in the pore solution of the cement matrix, increasing the tendency to precipitate more CSH gel [10]. 

Gypsum plays a key role in cement mixes when limestone of any grade is used as a supplementary cementitious material. Gypsum determines the set time and the strength gain of concrete [15] and exists in many forms, including anhydrous, dihydrate, and hemihydrate gypsum, with different solubility rates. The amount of gypsum to be used in limestone-based cement mixes must be specified because it affects not only the setting time but also the strength development and expansion. The minimum gypsum content required to control the setting time is around 2%. Two main observations due to the additions of gypsum to the limestone-based cement mixes are (i) an increase in the degree of the hydration of tricalcium silicate (C_3_S) due to the aluminate peak pushing beyond the silicate peak and (ii) an increase in the amount of ettringite crystals due to additional sulfate from the gypsum [16]. Furthermore, a suitable content of limestone and gypsum can have some positive influence on concrete properties, such as filler, nucleation, and chemical effects, as well as improving workability [17]. However, the optimal content of LGL–gypsum–cement blends still needs further study in concrete systems with a relatively low water/binder ratio and a high superplasticizer dose.

Limestone-based concrete mixes need a chemical superplasticizer due to the existing water scarcity problem in tropical countries with water shortages [18], further increasing the total cost of concrete and the environmental burden [19]. Superplasticizers, considered high-range water reducers, are often incorporated into such mixes to reduce water demand and thus achieve a higher solid content without eliminating consistency [20]. The addition of weakly reactive powders would result in the higher yield stress of fresh cement mixes and increased demand for plasticizers [21]. Kakali et al. [22] found that the setting time and consistency of cement mixes were reduced with increasing limestone additions to 35%. There is a significant reduction in the yield stress of cement mixes due to the addition of 15% limestone. These rheological characteristics of fresh concrete mixes are regulated by the fineness of the cement and the replacement levels [23]. The dose of superplasticizer depends on the type of cement, the shape, and the dimension of the cementitious materials, the water/binder ratio (w/b), the type of liquid admixture, the compatibility between cement and superplasticizer, as well as the temperature and humidity of the raw materials and the environment [24]. The compatibility and synergistic effect between the addition of LGL, gypsum, cement, and superplasticizer and the amount of water have very rarely been investigated.

This study aims to determine the optimal dose of superplasticizers based on polycarboxylate ether and sulfonated naphthalene formaldehyde required to obtain a maximum yield in cement mixes containing 35% LGL and 2% gypsum. The effects of the optimal doses of the superplasticizers on the various properties of LGL-based mortar samples were also examined by performing flow table, compressive strength, and shrinkage tests. The microstructure of these mortar samples at the end of 28 days was studied by conducting scanning electron microscopy (SEM) and X-ray diffraction (XRD) investigations. LGL is a leftover material in cement quarries, and its usage as a supplementary cementitious material requires more water, while the test findings of this study help to understand the impact of the dose, type, and properties of the superplasticizer on the performance of the cement mixes containing a higher volume of LGL and provide recommendations on the selection of superplasticizers that can produce maximum flow at lower doses.

## 2. Materials and Methods

### 2.1. Sample Preparation

**Figure 1:**
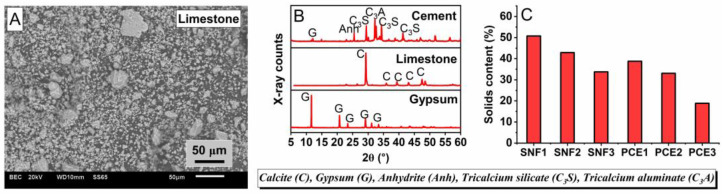
(**A**) SEM image of limestone, (**B**) XRD pattern of raw materials, and (**C**) solid content of different superplasticizers used in this study.

Ordinary Portland cement of 53 grade according to IS 12269 [25] was used in the preparation of different mortar samples. Other raw materials were low-grade limestone (LGL), gypsum, potable water, crushed granites derived from granite origin, and superplasticizers based on polycarboxylate ether (PCE) and sulfonated naphthalene formaldehyde (SNF). The SEM image in Figure 1 reveals the angular structure of the LGL sample. The raw material was mounted on a steel stub using a carbon adhesive and scanned with a JSM-7600-F SEM (JEOL, Tokyo, Japan) at a higher voltage to obtain information about the microstructure. The cementitious materials were obtained from a local cement manufacturer in India. The specific surface areas of LGL, gypsum, and cement reported by the manufacturer are 165, 140, and 310 m^2^/kg. The samples of these raw materials were sieved to a size lower than 75 μm and scanned with a Bruker model X-ray diffraction at a rate of 0.02° 2θ/min for a 2θ range between 5 and 80°. The main XRD peaks illustrated in Figure 1 confirm the main presence of tricalcium silicate, tricalcium aluminate, and calcite in cement and LGL. These samples were pelletized on a circular metal disc and scanned with a Rigaku model X-ray fluorescence (XRF) to obtain information on the chemical composition. Table 1 lists the XRF results in which the CaO content of cement was calculated to be 1.6 times higher than that of LGL. Their loss of ignition was also found by heating them in a hot air oven at 1000 °C, and subsequent weight losses were calculated. The loss of ignition (LOI) of LGL was very high, indicating the main presence of organic species. Crushed granite sand conforming to Zone II of IS 383 [26] was used in the preparation of mortar samples. It had a specific gravity of 2.58 and a water absorption of 2.27. These aggregate properties were determined according to IS 2386 [27]. PCEs and SNFs according to IS 9103 [28] were obtained from three different chemical suppliers, Sika, Fosroc, and BASF, and were designated PCE1, PCE2, PCE3, SNF1, SNF2, and SNF3. Their pH and relative density were found to fall within the range of 6.37–8.31 and 1.06–1.31, while the solid content is illustrated in Figure 1. These physical properties were found according to IS 9103 [28]. The optimal doses of each superplasticizer in different w/b ratios of 0.35, 0.4, 0.45, and 0.5 were determined in fresh paste samples containing 63% cement, 35% LGL, and 2% gypsum. The optimum dose information obtained from the experiments on fresh paste samples was then adopted in the preparation of mortar samples to validate the impact of different superplasticizers on the performance of cement mixes containing a higher volume of LGL.

Table 2 lists the details of the cement-based mortar mixture that was prepared in different w/b ratios and proportioned with the optimal doses of superplasticizers that resulted from the investigation of the fresh paste samples. The cementitious materials were mixed with crushed aggregates, water, and superplasticizers in a Hobart high-speed mixer for 3 min to cast samples of different sizes. Acrylic molds were used to prepare mortar samples that were demolded after 24 h of casting and stored in water to cure until the age of testing. Sample consolidation was performed using a vibrating table.

### 2.2. Isothermal Calorimetry and Vicat Apparatus

Approximately 30 g of fresh cement paste was loaded into the test channels of an I-Cal 8000 Calmetrix high-precision isothermal calorimeter according to ASTM C1702 [29]. Heat developments in this sample were recorded for up to 7 days at 25 °C and 65% relative humidity (RH). The initial and final setting time of the fresh cement paste was measured according to IS 4031 [30]. About 300 g of cement was added to 72 g of water and quickly mixed before filling the Vicat apparatus sample mold according to IS 5513 [31]. A 1 mm square needle was allowed to penetrate this fresh paste every 10 min until the apparatus index scale read approximately 5 mm from the bottom of the mold. Then, the annular needle was replaced to measure the final setting time. This needle was released every 30 min until it made an impression on the sample. An average of three measurements was calculated.

### 2.3. Mini Slump and Marsh Cone Tests

**Figure 2:**
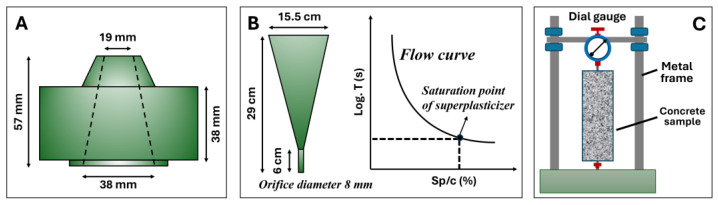
Details of the (**A**) mini-slump, (**B**) Marsh cone, and (**C**) shrinkage tests.

Consistency was evaluated using the mini-slump cone apparatus [32] of dimensions: diameter 19 mm, bottom diameter 38 mm, and height 57 mm. The mold was placed firmly on a flat horizontal plastic sheet, filled with paste, and compacted with a spatula. When the mold was full, the top surface was leveled and excess paste was removed. The mold was vertically removed, ensuring minimal lateral disturbance, and to avoid sample disturbance, the slumped sample was left to harden for 24 h. The diameter of the hardened base was measured at five locations around the outline and the mean was used to calculate the area of the base. A metal cone was used according to EN 445 [33] with an 8 mm diameter bottom nozzle. Around 1000 mL of fresh cement paste was placed in the Marsh cone, while the bottom orifice of the tapered cone was kept closed. The orifice was then opened, and the fresh paste was allowed to flow into a 1000 mL capacity graduated cylindrical flask, which was kept below. The time it took 1000 mL of the cement paste to flow out of the Marsh cone was recorded as the efflux time of the paste. Figure 2 illustrates the details of the mini-slump and Marsh cones.

### 2.4. Flow Table, Compressive Strength, and Shrinkage Tests

The fresh mortar sample was filled into the truncated cone and placed in the center of the flow table apparatus. The cone was removed leaving the fresh sample, which was dropped continuously for 15 times. The mortar flow is the resulting increase in the average base diameter of the mortar mass, measured in at least four diameters at equivalently spaced intervals expressed as a percentage of the original base diameter. The mortar sample of dimensions 70 × 70 × 70 mm^3^ was tested at the end of 28 days according to IS 516 [34] using a MATEST model servo-controlled compression test machine with a capacity of 3000 kN. The loading rate was maintained at 35 MPa/s. The load at failure divided by the cross-sectional area of the sample gives the compressive strength. Prismatic mortar samples of 25 × 25 × 285 mm^3^ size were used to perform the shrinkage test at 25 °C and 65% RH according to ASTM C157 [35]. The change in sample length was recorded at frequent intervals until the end of 28 days using a length comparator frame supported with a 1 μm precision dial gauge. This frame was calibrated using a standard invar bar. The shrinkage strain was calculated by subtracting the initial reading from the dial gauge and the readings taken at the subsequent intervals of t and then divided by the length of the sample. In these tests, a mean of four replicates was calculated.

### 2.5. Characterization Studies

Small mortar fragments were collected from the cube samples tested under compression after 28 days. Care was taken to exclude the aggregates from the mortar pieces obtained. They were dried at room temperature for a day, powdered with mortar and pestle, sieved to a size of less than 75 µm, and finally scanned for a 2θ range of 5–80° using an XRD at a scanning rate of 0.02°/min. The mortar chunks obtained from the crushed samples were dried at room temperature for a day, mounted onto steel stubs using a carbon adhesive, gold-coated for 60 s, and analyzed at 20 kV current under secondary electron mode using the SEM.

## 3. Results and Discussion

### 3.1. Heat of Hydration and Setting Time

**Figure 3:**
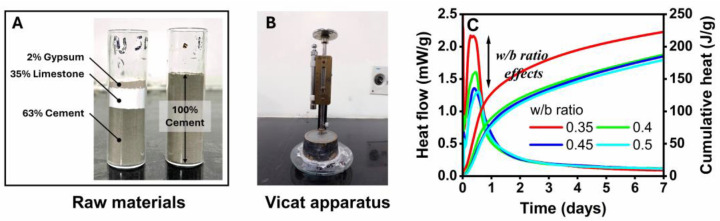
(**A**) Raw materials, (**B**) Vicat apparatus, and (**C**) isothermal calorimetry test results.

Figure 3 shows the isothermal calorimetry test results of the fresh paste samples containing 63% cement, 35% LGL, and 2% gypsum. These samples exhibited an exothermic reaction when water was added to them and exhibited the maximum amount of heat around 8 h after mixing. The heat release of these pastes was reduced with an increasing w/b ratio, indicating a slower increase in the concentration of calcium ions in the pore solution of these fresh samples at the beginning of age. LGL particles have little effect on the hydration of cement mixes [13]. Its nucleation effect accelerates the maximum hydration of C_3_S and generates more hydration products at an early age. However, the dilution effect of the LGL particles affects the hydration of C_3_S and reduces the formation of Ca(OH)_2_ [36]. When the LGL and cement particles are mixed together, the water film governs the space between them and has a relatively significant effect on the rate of hydration of the cement during the post-peak hydration period [37]. The cement mixtures in this study include 63% cement, 35% LGL, and 2% gypsum. This raw cement was made up of 97% clinker and 3% gypsum according to the manufacturer. Therefore, the total gypsum content in the paste samples was calculated to be 3.9%. The average initial and final setting times for these samples were determined to be 160 and 310 min, while those for the plain cement paste were 170 and 295 min. Although the gypsum content in the plain cement and LGL-based mixtures was relatively higher, the initial cement setting still improved to 6% in the presence of a larger volume of LGL.

### 3.2. Mini-Slump Flow and Saturation Dose

**Figure 4:**
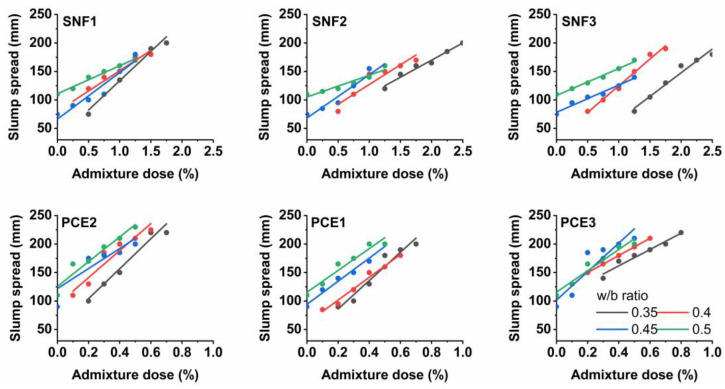
Results of the mini-slump tests.

**Figure 5:**
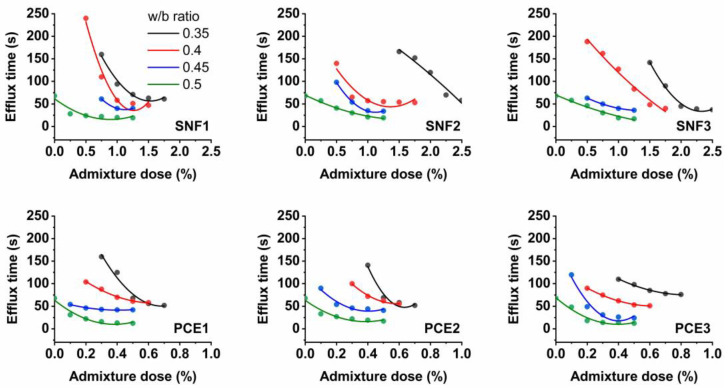
Results of the Marsh cone tests.

Figure 4 and Figure 5 show the results of the mini-slump and Marsh cone tests. The addition of SNF and PCE improved the slump flow of the paste mixes regardless of the w/b ratios used. They exhibited a mini-slump flow diameter of 200 mm when the dose of the superplasticizers was maintained above 1.5%. No slump flow was observed when the LGL-based cement pastes were made with w/b ratios lower than 0.4. They were sticky and stiff, and they needed a minimum dose of the superplasticizer of 0.5% to escape the test cone apparatus under gravity. The action of a superplasticizer is to prevent the flocculation of cement particles. The adsorption of SNFs leads to a decrease in the zeta potential and eventually causes (negative) charges on the cement particles. With the progress of hydration, the electrostatic charge decreases and the hydrating product flocculates [38]. The optimal amount of superplasticizer to be used in the design of the cement mix is determined by the flow curve. The efflux time in the Marsh cone decreases with an increase in the superplasticizer dosage. There is a saturation point of the superplasticizer dose beyond which there is no significant reduction in the efflux time. The results of the Marsh cone test showed that the efflux time of the fresh limestone-based cement pastes was reduced with an increase in the dose of SNF1, SNF2, and SNF3. The use of w/b ratios less than 0.4 resulted in a longer efflux time, but the fresh pastes containing low doses of such superplasticizers quickly drained out of the test apparatus under gravity at higher w/b ratios. 

The fresh limestone-based cement samples consumed less SNF1 to reach the saturation point in all the w/b ratios compared to those with SNF2 and SNF3. SNF1 has a higher solid content, which therefore influenced its saturation point in the limestone-based cement pastes. The active solid content of SNF1, SNF2, and SNF3 was 50.8%, 42.9%, and 33.8%, respectively. The efflux time of the limestone-based cement mixes was shorter when a minimum SNF dose of 1% was maintained, regardless of the w/b ratios used. No significant changes in the efflux time were observed when the fresh cement pastes contained doses of superplasticizer greater than 1%. PCE1, PCE2, and PCE3 are PCEs, and limestone-based cement pastes made with them also showed a rheological behavior like that occurring with SNFs. The use of PCEs in the limestone-based cement pastes resulted in a maximum slump flow diameter of 220 mm. At a 0.3 w/b ratio, the fresh limestone-based cement paste was able to escape the mini-slump cone apparatus when dosed with a minimum PCE dose of 0.2%. In general, SNFs work with the mechanism of lowering the zeta potential, leading to electrostatic repulsion. However, polymers with backbone and graft chains, such as PCE, cause the dispersion of cement grains by steric hindrance [39]. This phenomenon is related to the separation of superplasticizer molecules from each other because of bulky side chains. Steric hindrance is a more effective mechanism than electrostatic repulsion. The side chains, mainly polyethylene oxide, that extend on the surface of the cement particles migrate in water [40], and the cement particles are dispersed by the steric hindrance of the side chains [41]. In this study, the saturation points of PCE and SNF were determined using an analytical method proposed by da Silva et al. [42] and Gomes et al. [43]. The efflux time of the limestone-based cement paste was measured when the dose of the superplasticizer varied with respect to the cement content (Sp/c) and a graph illustrated in Figure 2 was plotted between the logarithm of time (T) and the percentage of the solid content of the superplasticizer. The saturation point of the superplasticizers (SPS) was then defined as the point within a 140° ± 10° interval. A descent exponential function shown in Equation (1) is used to define this graph between Log. T and Sp/c, where A_1_, t_1,_ and y_0_ are the constant parameters for each test curve [43]. From the function curve, it is possible to calculate its derivative and fit the curve to Equation (2) using data analysis and graphing software (e.g., OriginLab version 8.5), which automatically gives the coefficient values. When comparing the slope of the curve to −0.57, the value of ‘x’ was found, that is, the solid concentration of SP used per gram of cement at the saturation point. 

**Figure 6:**
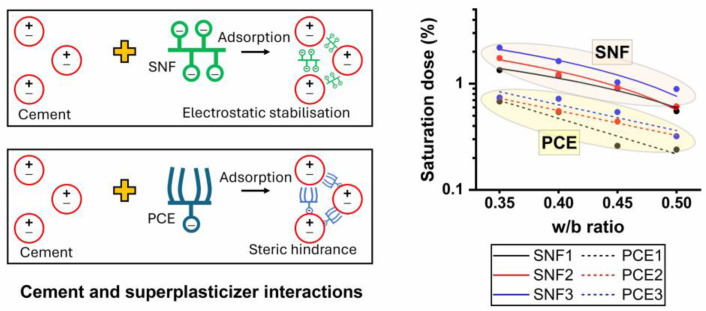
Saturation points of different superplasticizers obtained by conducting Marsh cone tests.

The optimal dose of SNFs and PCEs was found using the back-calculation technique and is shown in Figure 6. It was found that the SPS in the LGL-based cement pastes depended on the type of superplasticizer and the w/b ratios used. The optimal doses of both SNF and PCEs in the LGL-based cement pastes were found to decrease with an increase in the w/b ratios. When SNF3 was used in such pastes, its SPS in the w/b ratios of 0.35, 0.4, 0.45, and 0.5 was calculated to be 2.18, 1.63, 1.03, and 0.89%, respectively. The saturation point of the SNFs in the limestone-based cement pastes was almost 59% lower when the w/b ratio increased from 0.35 to 0.5. Similarly, to the plain cement mixes, the optimal doses of the SNFs in the limestone-based cement pastes were comparatively higher than the PCEs. The SPS in the w/b ratios of 0.35, 0.4, 0.45, and 0.5 was calculated to be 0.68, 0.54, 0.26, and 0.24%, respectively, when PCE1 admixture was used in the preparation of the limestone-based cement pastes. The dispersion of SNFs in cement mixes is caused by electrostatic repulsive forces, while both steric hindrance and electrostatic repulsion mechanisms govern the dispersion of PCEs in cement mixes. The optimal doses of these superplasticizers to be used in cement mixes depend on the content of active solids available in them. The degree of cement fluidity depends on the amount of superplasticizer adsorbed onto the surface of the cement particles. With increasing solid content, the amount of superplasticizer adsorbed on the cement particle increases. Both SNFs and PCEs are usually supplied as liquid formulations with an active solid content in the range of 30–40%. The behavior of superplasticizers is also a function of the structure and degree of polymerization. Side chains tend to extend from the adsorbed water-reducing admixture into the solution, and these side chains are anti-aggressive, and their steric hindrance significantly improves the fluidity of Portland limestone cement systems [44]. Increased fluidity decreases the efflux time and thus decreases the SPS values [45].

(1) $$f\left(x\right)=A_{1}e^{-\left(\frac{x}{t_{1}}\right)}+y_{0}$$

(2) $$\frac{dy}{dx}=-A_{1}\frac{e^{-\left(\frac{x}{t_{1}}\right)}}{t_{1}}=-0.57$$

### 3.3. Mortar Performance

**Figure 7:**
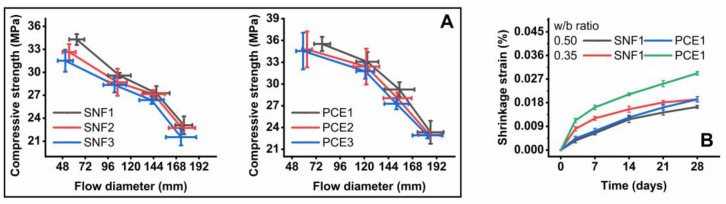
Results of the (**A**) flow table and compressive strength, and (**B**) shrinkage tests.

Figure 7 shows the flow table, compressive strength, and shrinkage test results of the mortar samples. The effect of the optimal doses of SNF and PCE on the flowability of fresh mortar mixes was determined at different w/b ratios. The fresh mortar samples were observed to be very sticky and stiff and did not flow in a 0.35 w/b ratio, but the samples made in other w/b ratios flowed in the fresh state. It was also observed that cement flow was reduced with a decrease in the w/b ratios regardless of the dose and type of the superplasticizers. The workability of fresh cement mixes rapidly decreases in a low w/b ratio. This effect is mainly due to the reduced distance between the cement particles, along with the constant consumption of free water through hydration [46]. The inclusion of a superplasticizer prevents (i) flocculation by adsorbing onto the surface of cement grains and (ii) the reduction in the entrapment of water in the flocculated structure, thus reducing the water demand to obtain the same fluidity in the cement matrix [47]. The flow diameter of the mortar samples was reduced up to three times when the w/b ratio was reduced from 0.5 to 0.35. There is a marginal decrease in the flowability of fresh mortar samples admixed with SNFs and PCEs when the active solid content in such superplasticizers is reduced. The addition of PCEs at lower doses was able to significantly improve the cement flow compared to SNFs. This beneficial effect is due to the dual working mechanisms of PCEs that include steric hindrance and electrostatic repulsion [48]. 

In this study, the compressive strength of the mortar samples tested after 28 days was reduced with increasing w/b ratios, regardless of the PCE and SNF used. However, the flowability and compressive strength of the mortar samples mixed with PCE were relatively higher than those containing SNF. The compressive strength of the mortar samples was reduced to almost 33% when the w/b ratio increased from 0.35 to 0.5. At a lower w/b ratio, the average compressive strength of the mortar samples admixed with SNF2 and PCE1 was found to be 34 and 36 MPa at the end of 28 days. There is no significant difference in the compressive strength of the mortar samples mixed with SNF and PCE when the active solid content of such superplasticizers is reduced. The shrinkage strain of these samples at the w/b ratios of 0.35 and 0.5 was measured over time. These mortar samples showed an increasing trend of shrinkage over time. At a lower w/b ratio, the shrinkage strain of the mortar samples admixed with SNF1 and PCE1 was calculated to be 0.029% and 0.019% at the end of 28 days. The addition of PCE reduced the mortar shrinkage by up to 34%. At lower w/b ratios, the use of PCEs marginally reduced the length change in the prismatic samples compared to those admixed with SNFs. However, this reduction in the mortar sample shrinkage due to SNF was relatively significant in the case of higher w/b ratios. The compressive strength of cement-based mixes decreases with increasing w/b ratio, and the content of raw cement is usually of minor influence, while its shrinkage and water absorption increase with the w/b ratio and cement content [49]. Sua-Iam and Makul [50] found a higher shrinkage in cement mixes containing a higher volume of rice husk ash, which absorbs more free water onto their surfaces while mixing. The use of PCE instead of SNF reduced this shrinkage, mainly because of their steric hindrance and electrostatic repulsion mechanisms.

### 3.4. Microstructure

**Figure 8:**
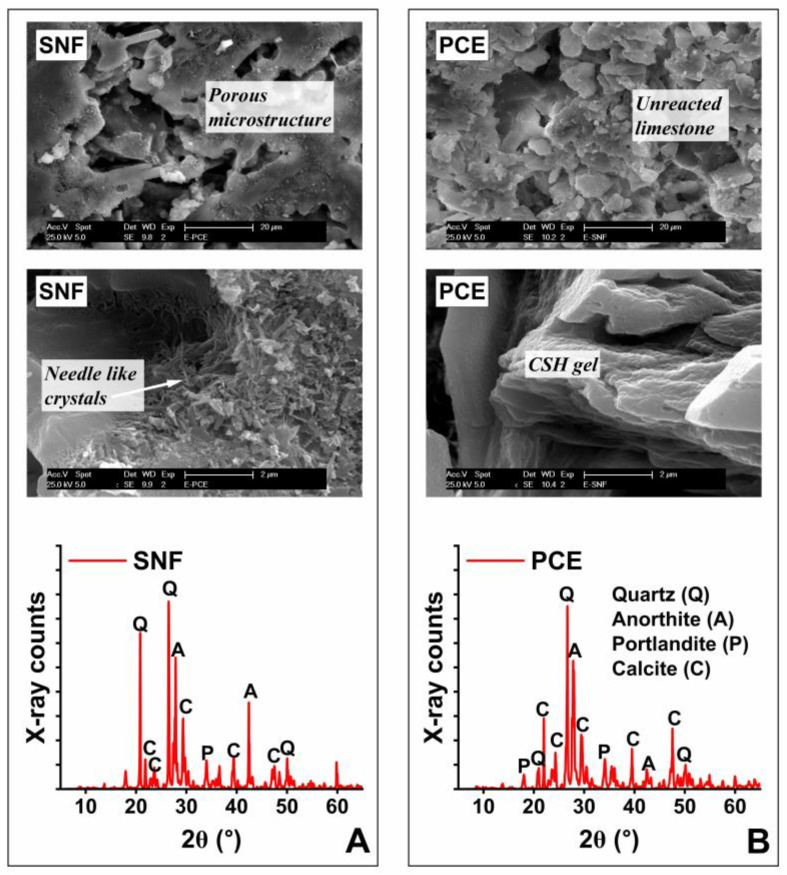
SEM and XRD results of the mortar samples admixed with (**A**) SNF and (**B**) PCE.

Figure 8 shows the SEM and XRD results. Unreacted LGL particles with rhombohedral structures were observed in the mortar samples admixed with PCE and SNF. Gypsum has an acicular structure and was not found in the sample microstructure as its amounts were relatively very low. The LGL particles improve the formation of the hydration rims of CSH surrounding C_3_S particles because they increase the rate of the hydration of C_3_S [51]. The increasing levels of LGL increased the formation of ettringite in the early ages [52]. The amount of ettringite then slowly decreased as the hydration proceeded. They did not observe any monosulfate [53]. To identify the crystalline phase and examine the crystal structure of specific phases existing in the mortar samples of this study, an XRD investigation was carried out on them. The major XRD peaks attributed to 9.1°, 10.7°, 18.1°, 20.7°, 21.8°, 26.5°, 27.8°, 29.4°, 34.1°, 39.3°, 42.5°, 48.5°, 50.2°, and 60.1° 2θ diffraction angle confirmed the availability of ettringite, gypsum, portlandite, quartz, calcite, C_3_S, C_2_S, and anorthite, respectively, in the mortar samples. The XRD method is only capable of identifying these crystalline reaction products and is unable to distinguish the CSH gel [54], which is because this major hydrate is poorly crystalline and amorphous. Anorthite could have formed because of the chemical reaction between calcium silicates and aluminate species in the mortar samples. The addition of LGL at the expense of gypsum can form what is known as carboaluminate instead of ettringite [55]. This often requires less water than in the case of gypsum [56].

## 4. Conclusions

In this study, cement mixes containing a higher volume of LGL were demonstrated. The effect of the type and properties of superplasticizers on their performance was examined by performing multiple tests. The heat developments in these mixes decreased with an increasing w/b ratio, and their flow was found to be zero when the w/b ratios were less than 0.4. The fresh mixes were able to flow only when the superplasticizer dose was greater than 0.5% in the case of SNF and 0.2% in the case of PCE. The optimal doses of SNF and PCE in the LGL-based cement mixes were found to decrease with increasing w/b ratios. The optimal doses of these superplasticizers were found to depend greatly on the content of the active solids available in them. The addition of SNF improved the cement flow by electrostatic repulsive forces, while such improvements were caused by both steric hindrance and electrostatic repulsion mechanisms in the case of adding PCE polymers. The compressive strength of the LGL-based mixes mixed with PCE was found to be relatively higher, indicating a lower agglomeration of cement particles during mixing. Early-age shrinkage was also found to be relatively lower in these mixes. Anorthite mineral-like compounds were observed in the microstructure of such mixes according to the results of the SEM and XRD studies. LGL as a supplementary cementitious material in concrete mixes requires more superplasticizers, and the findings of this study suggest the use of PCE admixture containing a higher active solid content to produce maximum flow and obtain a higher compressive strength.

## Figures and Tables

**Table 1:** Chemical composition of raw materials.

Sample (%)	CaO	SiO_2_	Al_2_O_3_	Fe_2_O_3_	MgO	Na_2_O	K_2_O	SO_3_	Cl	LOI
Cement	61.77	20.01	4.97	4.4	1.22	0.14	0.28	2.56	0.01	4.64
Limestone	39.27	15.77	3.37	0.43	1.56	0.07	0.17	0.24	-	39.12
Gypsum	29.81	3.34	1.35	0.49	1.33	-	-	43.85	0.01	19.82

**Table 2:** Details of the cement-based mortar mixture.

Cement (g)	Limestone (g)	Gypsum (g)	Sand (g)
630	350	20	2500

## Data Availability

The original contributions presented in the study are included in the article, further inquiries can be directed to the corresponding author.
